# Does growth hormone supplementation of in vitro fertilization/intracytoplasmic sperm injection improve cumulative live birth rates in women with poor embryonic development in the previous cycle?

**DOI:** 10.1186/s12958-024-01223-9

**Published:** 2024-05-07

**Authors:** Xitong Liu, Na Li, Dongyang Wang, Wen Wen, Li Tian, Hanying Zhou, Ben W. Mol, Juanzi Shi, Tao Wang

**Affiliations:** 1 The Assisted Reproduction Center, Northwest Women’s and Children’s Hospital, No. 73 Houzai Gate, Shaanxi Province, PO Box 710003, Xincheng District, Xi’an CityXi’an, China; 2 Translational Medicine Center, Northwest Women’s and Children’s Hospital, Xi’an, China; 3 Department of Obstetrics and Gynaecology, Monash Medical Centre, Monash University, Wellington Road, Clayton VIC 3800, Victoria, Australia; 4 School of Medicine, Medical Sciences and Nutrition, Aberdeen Centre for Women’s Health Research, University of Aberdeen, Aberdeen, UK

**Keywords:** Growth hormone, Cumulative live birth rate, IVF, Propensity score matching, Embryo quality

## Abstract

**Background:**

Growth hormone (GH) has been proposed as an adjunct in in vitro fertilization (IVF)/intracytoplasmic sperm injection (ICSI) cycles, especially in women with poor ovarian response. However, it is unclear whether GH supplementation is effective in women with poor embryonic development in the previous IVF cycle. The aim of this study was to evaluate the effectiveness of GH supplementation in IVF/ICSI cycles in women with poor embryonic development in the previous cycle.

**Methods:**

This is a retrospective cohort study from a public fertility center in China, in which we performed propensity score-matching (PSM) for female age and AFC in a ratio of 1:1. We compared the cumulative live birth rate per started cycle, as well as a series of secondary outcomes. We included 3,043 women with poor embryonic development in the previous IVF/ICSI cycle, of which 1,326 had GH as adjuvant therapy and 1,717 had not. After PSM, there were 694 women in each group.

**Results:**

After PSM, multivariate analyses showed the cumulative live birth rate to be significantly higher in the GH group than the control group [*N* = 694, 34.7% vs. *N* = 694, 27.5%, risk ratio (RR): 1.4 (95%CI: 1.1–1.8)]. Endometrial thickness, number of oocytes retrieved, number of embryos available, and number of good-quality embryos were significantly higher in the GH group compared to controls. Pregnancy outcomes in terms of birth weight, gestational age, fetal sex, preterm birth rate, and type of delivery were comparable. When we evaluated the impact of GH on different categories of female age, the observed benefit in the GH group did not appear to be significant. When we assessed the effect of GH in different AFC categories, the effect of GH was strongest in women with an AFC5-6 (32.2% versus 19.5%; RR 2.0; 95% CI 1.2–3.3).

**Conclusions:**

Women with poor embryonic quality in the previous IVF/ICSI cycles have higher rates of cumulative live birth with GH supplementation.

**Supplementary Information:**

The online version contains supplementary material available at 10.1186/s12958-024-01223-9.

## Introduction

In vitro fertilization (IVF) is the cornerstone of modern infertility treatment, with an average live birth rate of 30% per transfer, resulting in cumulative live birth rates as high as 70% per started cycle. Female age and subsequent poor oocyte quality, however, is the main limiting factor of IVF success. Indeed, poor embryo quality results in low success rates [1, 2]. Improvement of embryo quality is therefore likely to improve clinical outcomes.

Growth hormone (GH) has been reported to be able to enhance the functional mitochondria in oocytes [3]. In vitro studies have shown that GH plays an important role in the proliferation of the theca cells [4]. Theoreticality, exogenous GH acts on insulin-like growth factor (IGF) receptors of the ovaries to increase steroidogenesis and oocyte maturation [5, 6].

We previously showed co-treatment with GH in women with normal ovarian response with poor embryo quality could increase clinical pregnancy rate (64.78% vs. 59.33%) [7]. Several reviews have suggested that GH supplementation improves IVF outcomes in poor responders [8, 9]. While some studies have demonstrated that pre-treatment of GH could potentially enhance pregnancy, implantation, and live birth rates, others have refuted the efficacy of GH as an adjuvant in infertility treatment due to the lack of significant increase in live birth rates. A recent Cochrane review therefore suggested there was insufficient evidence regarding the effect of adjuvant GH for routine use in IVF [10].

In view of this evidence gap, we studied the effects of GH supplementation in women with poor embryonic quality in previous cycles.

## Materials and methods

### Study design

We performed a retrospective, single-center cohort study in the Assisted Reproductive Center of Northwest Women’s and Children’s Hospital, Xi’an, China. The study protocol was approved by the Ethics Committee of Northwest Women’s and Children’s Hospital (No. 2022007).

We studied women treated between January 2017 and December 2020. Women were eligible if they met the following criteria: (1) undergoing a second IVF/ICSI cycle with a failure to achieve pregnancy in the first attempt; (2) no top-quality embryos on day 3 (grade I or II) in the first cycle [11]; and (3) age 20–45 years old. Exclusion criteria were: (1) hyperthyroidism or hypothyroidism; (2) hyperplasia of mammary glands; (3) history of malignant tumor; (4) diabetes mellitus; (5) inclusion in this study in a previous cycle.

### Ovarian stimulation protocols

Ovarian stimulation could be with GnRH agonist or GnRH antagonist protocols, as has been described in detail elsewhere [12]. Briefly, for the GnRH agonist protocol, pituitary down-regulation began during the mid-luteal phase of the previous menstrual cycle with the GnRH agonist at a dose of 0.1–0.05 mg/day for 14 days. Recombinant follicle-stimulating hormone (rFSH) was started at 150–225 IU/day for ovarian stimulation. The dose of rFSH could be adjusted up to 300 IU/day based on ovarian response. Recombinant luteinizing hormone (rLH) could be added at the discretion of the treating physician.

For the GnRH antagonist protocol, rFSH was started on day 2 of the menstrual cycle, with similar doses of rFSH as the GnRH agonist protocol. GnRH antagonist, 0.25 mg/day was started when the dominant follicle reached 12–14 mm. When two or more follicles reached 17 mm, human chorionic gonadotropin (hCG) was given at a dose of 4,000 to 10,000 IU, and oocyte retrieval was performed 36 h later.

### Growth hormone supplementation

The choice to use GH was based on the preference of the woman and her treating physician. Women in the GH group received 2 IU recombinant human GH (Jintropin, Gensci, China) daily, from the initial day of pituitary down-regulation for the GnRH agonist protocol or day 2 of the previous menstrual cycle for the GnRH antagonist protocol until the day of the hCG trigger. Otherwise, treatment of the groups was similar.

### Embryo quality assessment

Embryo quality was assessed on day 3 at 72 h after oocyte retrieval. Embryos were scored according to a combination of blastomere number, blastomere size and fragmentation [13]. Briefly, embryos with 8–10 blastomeres, even homogeneous blastomeres < 10% cytoplasmic fragmentation were classified as grade I - embryos; embryos with 6–7 or > 10 blastomeres with even homogeneous blastomeres of no cytoplasmic fragmentation; or embryos with 8–10 blastomeres with even homogeneous blastomeres of 10%-20% cytoplasmic fragmentation were classified as grade II - embryos; embryos with 4–5 blastomeres with uneven and non-homogeneous blastomeres with 20%-50% cytoplasmic fragmentation were classified as grade III - embryos; embryos with fewer than 4 blastomeres with uneven and non-homogeneous blastomeres with > 50% cytoplasmic fragmentation were classified as grade IV—embryos (Supplementary Table 1). Only embryos classified as grade I, II, and III were available for transfer.

Embryos of grade I and II were regarded as top-quality embryos. For women with more than four top-quality cleavage embryos, all embryos were cultured to the blastocyst stage. A maximum of two embryos were transferred per transfer. The remaining embryos were frozen for future use. Women who were at risk of ovarian hyperstimulation syndrome (OHSS), women who presented with hydrosalpinx, and women who had high progesterone levels on hCG trigger day had frozen-thawed embryo transfer.

### Luteal phase support and pregnancy confirmation

Luteal support was given with 600 mg of vaginal progesterone and 30 mg oral progesterone daily from the day of oocyte retrieval in the fresh cycle or the day of embryo transfer in the frozen-thawed embryo transfer cycle. A pregnancy tests using serum β-hCG was performed 14 days after embryo transfer. In case of a positive pregnancy test, transvaginal ultrasound was performed 5 weeks after embryo transfer to determine the number of gestational sacs and the fetal heartbeat.

### Outcome measures

The primary outcome was cumulative live birth, defined as a live birth > 24 weeks of gestation, following the use of all fresh and frozen embryos derived from a single ovarian stimulation cycle. Secondary outcomes were biochemical pregnancy, clinical pregnancy, ongoing pregnancy, multiple pregnancy, miscarriage (defined as a pregnancy failure that occurs before 24 completed weeks of pregnancy) and ectopic pregnancy. We also assessed number of embryos, embryo quality and number of embryos available.

For women achieving live birth, we reported birth weight, fetal sex, gestational age at delivery in weeks, preterm birth (defined as delivery before 37 completed weeks of pregnancy) and type of delivery. All women in the study were followed-up until 2 years after oocyte retrieval.

### Statistical analysis

**Fig. 1:**
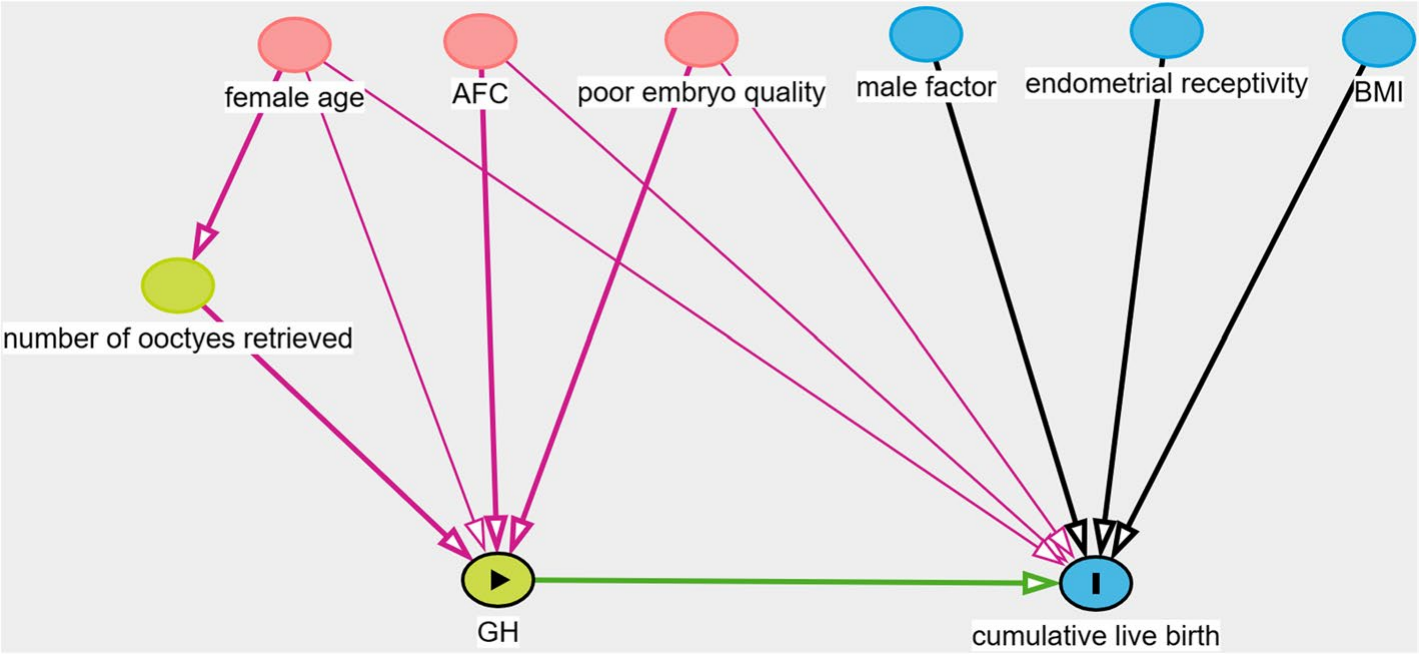
Directed acyclic graphs in identification selection of covariates

Propensity score matching (PSM) was performed to match the baseline characteristics of GH and control groups. Confounding was assessed by utilizing prior knowledge with the aid of directed acyclic graphs (DAG) (Fig. 1). The subsequent covariates were contemplated for incorporation in the ultimate model to match the GH group to the control group with a 1:1 ratio: female age, AFC, and embryo quality in previous cycle.

Categorical variables were expressed as percentages and were compared using the chi-square test or Fisher’s exact test. Continuous variables were expressed as mean ± SD and were compared using Student’s *t* test and the Mann–Whitney *U* test. Multivariable logistic regression analyses were used to determine the adjusted risk ratios (aRR) and 95% confidence intervals (CIs) for dichotomous outcomes. In the multivariable analyses we adjusted for female age, male age, basal FSH, AFC, body mass index (BMI), infertility duration, and infertility type. Subgroup analysis was performed with quartiles in different female age groups and AFC groups before and after PSM. Subgroup factor (female age and AFC) in the Poisson regression model was used to test the treatment-covariate interaction.

Data were analyzed with the use of the statistical packages R (The R Foundation; http://www.r-project.org.version 3.4.3) and Empower (R) (http://www.empowerstats.net/en/, X&Y solutions, inc. Boston, Massachusetts). A P-value < 0.05 was supposed to indicate statistical significance.

## Results

**Fig. 2:**
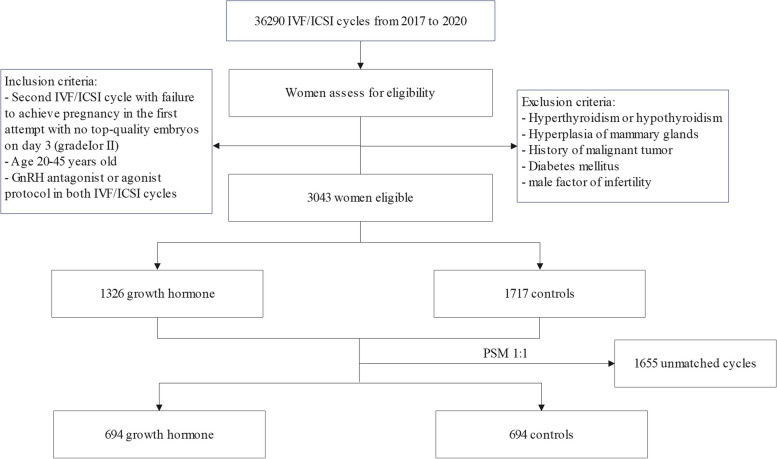
Flowchart of study cohort

**Fig. 3:**
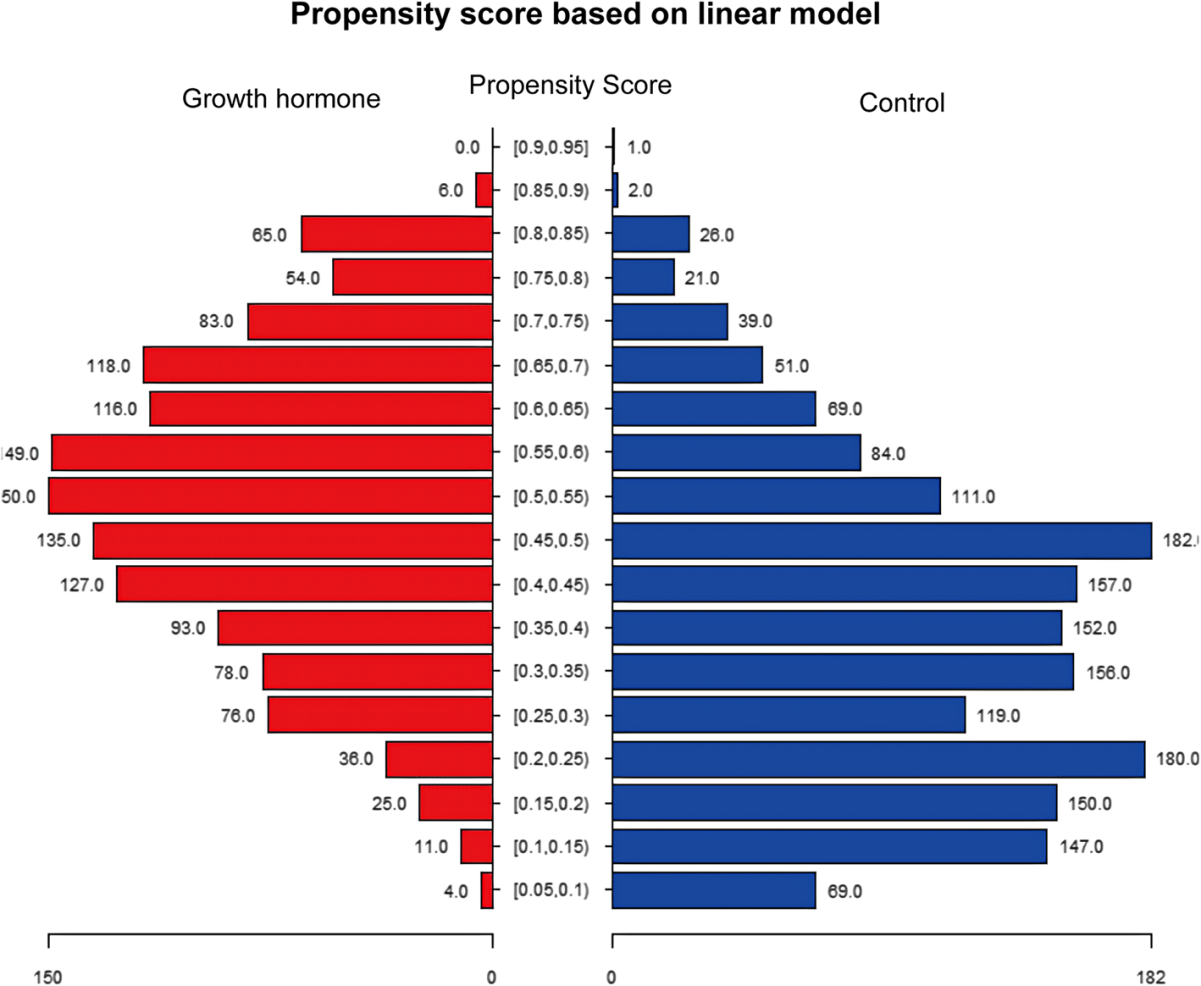
Propensity score in two groups

Between January 2017 and December 2020, 36,290 IVF/ICSI cycles were performed in our center. After assessing for eligibility, 3,043 women had a previous cycle without top-quality embryos on day 3 (grade I or II) and were eligible for the study. Of these women, 1,326 women were treated with GH and while 1,717 women did not use GH (Fig. 2). After PSM, 694 women treated with GH (intervention) could be matched to 694 women treated with regular IVF without GH supplementation (control). Propensity score in the two groups was shown in Fig. 3.

Table 1 presents the demographic characteristics of women before and after PSM. After PSM, there was no statistically significant difference in demographic characteristics between the two groups, particularly regarding the embryo quality in previous cycle.

**Table 1:** Demographic characteristics of women in the growth hormone and control groups

Characteristic	Before propensity score matching			After propensity score matching		
	**Growth hormone (*n* = 1,326)**	**Control (*n* = 1,717)**	***P* value**	**Growth hormone (*n* = 694)**	**Control (*n* = 694)**	***P* value**
Female age (y)	32.3 ± 4.7	34.6 ± 5.8	< 0.001	33.0 ± 4.8	33.0 ± 4.8	0.99
AFC	10.5 ± 5.8	6.9 ± 5.3	< 0.001	8.5 ± 5.1	8.5 ± 5.1	0.99
Male age (y)	33.9 ± 5.1	36.6 ± 6.5	< 0.001	34.6 ± 5.3	35.1 ± 5.4	0.08
Basal FSH (IU/L)	8.8 ± 5.8	10.8 ± 7.5	< 0.001	9.2 ± 6.2	9.9 ± 6.5	0.12
BMI (kg/m^2^)	22.5 ± 3.2	22.7 ± 3.2	0.11	22.5 ± 3.2	22.6 ± 3.3	0.31
Infertility duration (y)	4.0 ± 2.9	4.0 ± 3.4	0.05	4.0 ± 2.9	4.0 ± 3.1	0.89
Primary infertility	665 (50.2%)	745 (43.4%)	< 0.001	334 (48.1%)	331 (47.7%)	0.87
Oocytes retrieved previous cycle	5.9 ± 4.4	5.5 ± 3.6	0.44	5.8 ± 4.1	5.9 ± 4.1	0.69
Embryo quality in previous cycle			< 0.001			0.99
Grade III only	39 (2.9%)	248 (14.5%)		17 (2.4%)	17 (2.4%)	
Grade III + IV	995 (75.0%)	831 (48.4%)		179 (25.8%)	179 (25.8%)	
Grade IV only	292 (22.0%)	637 (37.1%)		498 (71.8%)	498 (71.8%)	

*FSH* follicle stimulating hormone, *AFC* antral follicle count, *BMI* body mass index, *IVF* in vitro fertilization

Endometrium was significantly thicker in the GH group (10.9 ± 2.7 versus 10.2 ± 3.1 mm, *p*-value < 0.001) (Table 2). Also, the number of oocytes retrieved (7.6 versus 6.6), the number of embryos available (3.3 versus 2.9), and number of good-quality embryos (1.8 versus 1.5) were higher after the use of GH.

**Table 2:** Characteristics in IVF cycles of two groups before and after propensity score matching

Characteristic	Before propensity score matching			After propensity score matching		
	**Growth hormone (*n* = 1,326)**	**Control (*n* = 1,717)**	***P* value**	**Growth hormone (*n* = 694)**	**Control (*n* = 694)**	***P* value**
Protocol			0.509			0.67
GnRH agonist	922 (53.70%)	728 (54.90%)		367 (52.9%)	359 (51.7%)	
GnRH antagonist	795 (46.30%)	598 (45.10%)		327 (47.1%)	335 (48.3%)	
Change in protocol after IVF failure	448 (33.8%)	547 (31.9%)	0.26	237 (34.1%)	232 (33.4%)	0.78
Total gonadotropin dosage (IU)	2541.2 ± 944.1	2540.6 ± 1142.0	0.63	2629.3 ± 968.6	2633.6 ± 1075.5	0.94
Total gonadotropin duration (days)	10.1 ± 2.5	9.4 ± 3.0	< 0.01	10.0 ± 2.6	9.8 ± 2.6	0.15
E_2_ level on hCG day	3202.4 ± 2452.7	2164.2 ± 2093.7	< 0.01	2763.1 ± 2148.0	2675.1 ± 2287.8	0.46
Endometrial thickness (mm)	11.2 ± 2.6	9.7 ± 3.1	< 0.01	10.9 ± 2.7	10.2 ± 3.1	< 0.01
Number of oocytes retrieved	9.0 ± 5.6	5.4 ± 5.1	< 0.01	7.6 ± 5.0	6.6 ± 5.2	< 0.01
Fertilization type			< 0.01			0.17
IVF	677 (51.9%)	889 (55.6%)		367 (54.0%)	337 (51.0%)	
ICSI	590 (45.3%)	694 (43.4%)		297 (43.7%)	315 (47.7%)	
IVF + ICSI	37 (2.8%)	15 (0.9%)		16 (2.4%)	9 (1.4%)	
Number of embryos available	3.7 ± 3.0	2.5 ± 2.5	< 0.01	3.3 ± 2.8	2.9 ± 2.8	< 0.01
Number of good-quality embryos	2.0 ± 2.3	1.3 ± 1.8	< 0.01	1.8 ± 2.1	1.5 ± 2.0	< 0.01
Number of transfers	1.2 ± 0.8	1.0 ± 0.7	< 0.01	1.2 ± 0.7	1.1 ± 0.8	0.27
Freeze-all cycles	306 (23.1%)	391 (22.8%)	0.84	161 (23.2%)	154 (22.2%)	0.65
Embryo type of first transfer			< 0.01			0.67
Cleavage embryos	807 (74.0%)	1039 (80.3%)		419 (75.4%)	416 (76.5%)	
Blastocyst embryos	283 (26.0%)	255 (19.7%)		137 (24.6%)	128 (23.5%)	

*IVF* in vitro fertilization, *ICSI* intracytoplasmic sperm injection

The cumulative live birth rate in the GH was significantly higher after PSM than control [34.7% vs. 27.5%, RR: 1.4, 95% confidence interval (95%CI) (1.1–1.8)] (Table 3). Secondary outcomes including live birth of first transfer (24.8% vs. 18.7%, RR: 1.4 (1.1, 1.8)), biochemical miscarriage (44.1% vs. 35.2%, RR: 1.5 (1.2–1.8)), clinical pregnancy (40.5% vs. 32.1%, RR: 1.4 (1.1–1.8)), and ongoing pregnancy (34.7% vs. 27.7%, RR: 1.4 (1.1–1.8)) were all higher after use of GH. For women achieving live birth, birth weight of singleton and twins, gestation delivery in weeks, fetal sex of singleton and twins, type of delivery were comparable.

**Table 3:** Comparisons of cumulative clinical outcomes before and after propensity score matching

**Characteristic**		**Before propensity score matching**				**After propensity score matching**		
	**Growth hormone (*n* = 1,326)**	**Control (*n* = 1,717)**	**aRR (95%CI)**^**a**^	***P* value**	**Growth hormone (*n* = 694)**	**Control (*n* = 694)**	**aRR (95%CI)**^**a**^	***P* value**
Cumulative live birth	499 (37.6%)	390 (22.7%)	1.5 (1.2, 1.7)	< 0.01	241 (34.7%)	191 (27.5%)	1.4 (1.1, 1.8)	< 0.01
Live birth of first transfer	325 (24.51%)	277 (16.13%)	1.4 (1.2, 1.7)	< 0.01	172 (24.8%)	130 (18.7%)	1.4 (1.1, 1.8)	< 0.01
Biochemical pregnancy	624 (47.1%)	521 (30.3%)	1.5 (1.3, 1.8)	< 0.01	306 (44.1%)	244 (35.2%)	1.5 (1.2, 1.8)	< 0.01
Clinical pregnancy	578 (43.6%)	472 (27.5%)	1.50 (1.3, 1.8)	< 0.01	281 (40.5%)	223 (32.1%)	1.4 (1.1, 1.8)	< 0.01
Ongoing pregnancy	503 (37.9%)	393 (22.9%)	1.5 (1.3, 1.8)	< 0.01	241 (34.7%)	192 (27.7%)	1.4 (1.1, 1.8)	< 0.01
Twin pregnancy	99 (19.5%)	55 (13.6%)	1.5 (1.0, 2.1)	0.05	50 (20.5%)	31 (15.8%)	1.4 (0.8, 2.3)	0.21
Miscarriage	80 (6.0%)	85 (5.0%)	1.2 (0.9, 1.7)	0.20	40 (5.8%)	34 (4.9%)	1.2 (0.8, 2.0)	0.47
Ectopic pregnancy	5 (0.4%)	2 (0.1%)	3.4 (0.6, 19.0)	0.17	3 (0.4%)	1 (0.1%)	3.0 (0.3, 30.7)	0.62
Birth weight (kg)								
Singleton								
Mean	3.3 ± 0.6	3.3 ± 0.5	NA	0.20	3.3 ± 0.5	3.3 ± 0.5	NA	0.25
Number of observations	408	351			173	132		
Low birth weight	2.0 ± 0.3	2.1 ± 0.4	NA	0.36	2.1 ± 0.3	2.2 ± 0.3	NA	0.64
Macrosomic infants	4.2 ± 0.3	4.2 ± 0.2	NA	0.61	4.2 ± 0.3	4.2 ± 0.1	NA	0.60
Twins								
Mean	2.5 ± 0.4	2.4 ± 0.4	NA	0.68	2.5 ± 0.4	2.5 ± 0.4	NA	0.89
Number of observations	99	55			50	31		
Low birth weight	2.1 ± 0.4	2.1 ± 0.3	NA	0.92	2.1 ± 0.4	2.1 ± 0.3	NA	0.92
Macrosomic infants	NA	NA	NA	NA	NA	NA	NA	NA
Gestation delivery in weeks	38.3 ± 2.1	38.4 ± 2.1	NA	0.49	38.5 ± 1.9	38.3 ± 2.1	NA	0.29
Fetal sex								
Singleton			NA	0.81			NA	0.52
Female	158 (48.0%)	124 (49.0%)			88 (50.9%)	72 (54.5%)		
Male	171 (52.0%)	129 (51.0%)			85 (49.1%)	60 (45.5%)		
Twins			NA	0.59			NA	0.89
Female twins	29 (29.3%)	12 (21.8%)			12 (24.0%)	6 (19.4%)		
Male twins	26 (26.3%)	15 (27.3%)			15 (30.0%)	10 (32.3%)		
Boy-girl twins	44 (44.4%)	28 (50.9%)			23 (46.0%)	15 (48.4%)		
Preterm birth	91 (18.2%)	63 (16.2%)	1.19 (0.83, 1.72)	0.35	35 (14.5%)	34 (17.8%)	0.8 (0.5, 1.4)	0.36
Type of delivery			NA	0.12			NA	0.29
Vaginal	101 (20.2%)	96 (24.6%)			49 (20.3%)	47 (24.6%)		
Cesarean section	398 (79.8%)	294 (75.4%)			192 (79.7%)	144 (75.4%)		

*aOR* adjusted odds ratio, *CI* confidence interval, *NA* not available/applicable

^a^Adjusted for female age, male age, basal FSH, AFC, BMI, infertility duration, and infertility type

When we evaluated the impact of GH on different categories of female age, the observed benefit in the GH group did not appear to be significant (Table 4). When we assessed the effect of GH in different AFC categories, the effect of GH was strongest in women with an AFC5-6 (32.2% versus 19.5%; RR 2.0; 95% CI 1.2–3.3).

**Table 4:** Cumulative live birth of women stratified according to female age and AFC after propensity score matching

Subgroup	Before propensity score matching				Subgroup	After propensity score matching			
	**Growth hormone (*n* = 1326)**	**Control (*n* = 1717)**	**Growth hormone vs control RR (95%CI)**	***P* for interaction**		**Growth hormone (*n* = 694)**	**Control (*n* = 694)**	**Growth hormone vs control RR**	***P* for interaction**
**Female age**				0.57	**Female age**				0.93
Quartile 1 (21–29)	181 (48.9%)	111 (32.8%)	1.5 (1.3, 1.8)		Quartile 1 (23–29)	76 (47.2%)	59 (36.6%)	1.5 (1.0, 2.4)	
Quartile 2 (30–32)	145 (36.9%)	103 (27.0%)	1.4 (1.1, 1.7)		Quartile 2 (30–31)	56 (38.6%)	47 (32.4%)	1.3 (0.8, 2.1)	
Quartile 3 (33–36)	131 (38.2%)	117 (28.1%)	1.4 (1.1, 1.7)		Quartile 3 (32–35)	71 (35.5%)	54 (27.0%)	1.5 (1.0, 2.3)	
Quartile 4 (> 36)	42 (19.1%)	59 (10.2%)	1.9 (1.3, 2.8)		Quartile 4 (> 35)	38 (20.2%)	31 (16.5%)	1.3 (0.8, 2.2)	
**AFC**				0.31	**AFC**				0.14
Quartile 1 (1–3)	19 (19.4%)	59 (11.6%)	1.7 (1.0, 2.6)		Quartile 1 (1–4)	32 (21.1%)	20 (13.2%)	1.8 (1.0, 3.2)	
Quartile 2 (4–6)	90 (30.5%)	107 (20.2%)	1.5 (1.2, 1.9)		Quartile 2 (5–6)	48 (32.2%)	29 (19.5%)	2.0 (1.2, 3.3)	
Quartile 3 (7–10)	123 (33.2%)	108 (29.8%)	1.1 (0.9, 1.4)		Quartile 3 (7–10)	73 (34.4%)	74 (34.9%)	1.0 (0.7, 1.5)	
Quartile 4 (> 10)	267 (47.4%)	116 (36.7%)	1.3 (1.1, 1.5)		Quartile 4 (> 10)	88 (48.6%)	68 (37.6%)	1.6 (1.0, 2.4)	

*AFC* antral follicle count

## Discussion

In this retrospective matched cohort study, we found that women with poor embryonic development in the previous cycle had an 8% higher cumulative live birth rate if they used GH in the new cycle. There were also more oocytes retrieved and more good quality embryos available after the treatment with GH.

Our present work has several strengths. Firstly, our study reports cumulative live birth rate extends the application of GH to an improvement of embryo quality. Secondly, we had a large sample size and PSM was conducted to control the potential confounders which might have effects on the outcomes. Comparisons were not only performed after PSM but were also explored before PSM.

The main limitation of our study is that, due to its retrospective nature, though PSM was performed, individual differences may still have existed, possibly affecting the results. Thus, further randomized controlled trials on GH co-treatment in women with poor embryo quality in the previous IVF/ICSI cycle are needed.

GH can affect oocyte and folliculogenesis via insulin-like growth factor 1 (IGF-1) or by the direct action of GH [9]. GH could improve ovarian response to gonadotropin via IGF-1, increasing oocyte competence by improving the mitochondrial activity of oocytes and possibly increasing the DNA repair capacity in oocytes [14–17]. The mitochondrial DNA in cumulus granulosa cells is proven to be positively associated with embryo development competence [18, 19]. GH also plays important antioxidant functions in oocytes [3] and could decrease reactive oxygen species (ROS) production associated apoptosis and activate the PI3K/Akt signaling pathway in granulosa cells [20].

The most recent Cochrane review identified 55 randomized studies of growth hormone as an adjunct to IVF, of which 39 studies were not used for the review but classified as waiting further information [10, 21]. Among 16 remaining studies, the effect of GH was estimate to be odds ratio (OR) 1.32, 95% CI 0.40 to 4.43. It was inconclusive to ascertain the effectiveness of GH supplementation.

Our previous study already suggested an effect of GH co-treatment in improving clinical pregnancy in women with a normal ovarian response [7]. Poor embryo quality driven by increased maternal age has a detrimental effect on clinical outcomes [22]. The number of clinical interventions to overcome poor embryo quality driven by maternal age are limited, including pretreatment with coenzyme Q10, melatonin, and artificial oocyte activation. In fact, IVF with oocyte donation is the only treatment that overcomes the detrimental impact of maternal age, albeit at the expense of transferring the use of the own genetic material of the woman.

It can be speculated that GH supplementation may also benefit women with poor embryo quality in other subgroups of ovarian reserve. Women of different ages and ovarian reserve can suffer from poor embryo quality, however, which subgroup of women could benefit from GH supplementation is still not clear. As co-treatment of GH is expensive and beyond indication, it is, therefore, essential to justify the potentially effective patients who may benefit from it, by improving the cumulative live birth rate. Randomized controlled trials are needed to confirm the findings.

## Conclusions

Our results suggest that women with poor embryonic development in the previous cycle could benefit from GH supplementation.

### Supplementary Information

**Supplementary Material 1.**

## Data Availability

No datasets were generated or analysed during the current study.

## Abbreviations

GH: Growth hormone
IVF: In vitro fertilization
ICSI: Intracytoplasmic sperm injection
PSM: Propensity score-matching
RR: Risk ratio
IGF: Insulin-like growth factor
rFSH: Recombinant follicle-stimulating hormone
rLH: Recombinant luteinizing hormone
hCG: Human chorionic gonadotropin
OHSS: Ovarian hyperstimulation syndrome
AFC: Antral follicle count
CI: Confidence interval
BMI: Body mass index
IGF-1: Insulin-like growth factor 1
ROS: Reactive oxygen species
OR: Odds ratio
