# Supplementary material for: Does growth hormone supplementation of in vitro fertilization/intracytoplasmic sperm injection improve cumulative live birth rates in women with poor embryonic development in the previous cycle?
Source: Reprod Biol Endocrinol. 2024 May 7;22:53. doi: 10.1186/s12958-024-01223-9 (PMC11075314; doi:10.1186/s12958-024-01223-9)
Supplement: Supplementary file 1 — Supplementary Material 1. [file 12958_2024_1223_MOESM1_ESM.docx]

**Supplementary Table 1** Embryo quality assessment

|  | **Blastomere number** | **Blastomere size** | **Cytoplasmic fragmentation** |
| --- | --- | --- | --- |
| GradeⅠ | 8-10 | even homogeneous blastomeres | <10% |
| GradeⅡ | 6-7 or >10 | even homogeneous blastomeres | 10%-20% |
| Grade Ⅲ | 4-5 | uneven and non-homogeneous blastomeres | 20%-50% |
| Grade Ⅳ | <4 | uneven and non-homogeneous blastomeres | >50% |
